# Comparing emotion inferences from dogs (*Canis familiaris*), panins (*Pan troglodytes*/*Pan paniscus),* and humans (*Homo sapiens*) facial displays

**DOI:** 10.1038/s41598-022-16098-2

**Published:** 2022-08-01

**Authors:** S. Kezia Sullivan, Ahyoung Kim, Lucio Vinicius Castilho, Lasana T. Harris

**Affiliations:** 1 Department of Experimental Psychology, University College London, London, England; 2 Department of Anthropology, University College London, London, England; 3 Department of Anthropology, University of Zürich, Zürich, Switzerland

**Keywords:** Psychology, Anthropology

## Abstract

Human beings are highly familiar over-learnt social targets, with similar physical facial morphology between perceiver and target. But does experience with or similarity to a social target determine whether we can accurately infer emotions from their facial displays? Here, we test this question across two studies by having human participants infer emotions from facial displays of: dogs, a highly experienced social target but with relatively dissimilar facial morphology; panins (chimpanzees/bonobos), inexperienced social targets, but close genetic relatives with a more similar facial morphology; and humans. We find that people are more accurate inferring emotions from facial displays of dogs compared to panins, though they are most accurate for human faces. However, we also find an effect of emotion, such that people vary in their ability to infer different emotional states from different species’ facial displays, with anger more accurately inferred than happiness across species, perhaps hinting at an evolutionary bias towards detecting threat. These results not only compare emotion inferences from human and animal faces but provide initial evidence that experience with a non-human animal affects inferring emotion from facial displays.

## Introduction

The human ability to infer other species' (hereafter ‘animals’) mind is well documented^1^. When humans infer other human minds, they can integrate statistical information about the context, target, and prior experiences (first-person or vicarious) to test models or hypotheses about how the target might think or feel^2,3^. Alternatively, they can simulate what the other is thinking or feeling and use such self-generated interoceptive information to guide the inference^4,5^. These accounts of social cognition hold implications for the inference of emotions from facial displays; either recognition through learned or *experienced* associations with contextual information^6^, or automatic recognition through covert imitation and mirror neuron activity^7^ due to perceived *similarity* of the perceiver with the target. However, the question remains whether either strategy is used when inferring the minds of agents beyond humans such as animals. Here, we test whether inferring emotions from facial displays depends on experience with or similarity to an animal target.

We compare emotion inferences from dogs (*Canis familiaris*), bonobos/chimpanzees (*Pan paniscus/Pan troglodytes,* hereafter referred to as *panins*), and humans (*Homo sapiens*) facial displays. We employ dogs and panins as animal targets due to the shared genetic ancestry and facial morphology in the case of the latter^8,9^, and shared environment in the case of the former^10^, to differentiate between the roles of morphological similarities and experience when inferring emotions from facial display. Both species have complex social lives^11,12^, with rules that must be obeyed if an individual is to be accepted by a group. As such, it is expected that they will communicate internal states with facial displays during social interactions^13^. Moreover, people’s gaze patterns when viewing animal and human faces are similar; particular attention is paid to the eye and mouth areas, the moving parts of the face^14^. Further, although the fusiform face area in the medial temporal lobe of the brain is most strongly activated by human faces, it also responds to animal faces^15^.

Dogs are often considered human’s closest animal companion; humans have interacted with wolves from as early as the Middle Pleistocene period and tamed these wolf pups, who later became the precursors of domesticated dogs^11^. Further, many people live with dogs, and therefore have extensive experience with dog’s emotional responses and corresponding facial displays. This makes dogs a highly experienced animal. There is a wealth of research demonstrating ways that the symbiotic relationship between dogs and humans may have led to adaptations in the social functioning of both species; Humans can accurately categorise the facial displays of dogs^16^, and dogs can accurately categorise both the facial displays of dogs and humans^17^. Domestication affected dogs’ facial muscle anatomy to facilitate communication with humans^18^. Experience with dogs also has an effect; dog professionals are more accurate at inferring emotions from dog facial displays and behaviour^16^, and people with personal or cultural experiences with dogs accurately infer emotions from their facial displays earlier in life^19^.

In contrast, panins are closely genetically related to humans, and share similar facial morphology and social hierarchies^9^ making them a highly similar animal to humans. Although panins have similar facial muscles to humans, they do not express emotion in facial displays the same way humans do. For instance, bare teeth in humans (i.e., smile) may indicate happiness, but to panins, it is a signal of safety^20^. Panins also have a measure of voluntary control over their facial displays^21^—differentiating them from other mammals such as dogs that cannot control their facial displays, highlighting the similarity between chimpanzee and human emotion regulation. Panins use facial displays to communicate with each other and maintain social bonds^22^. However, humans rarely encounter panins, instead viewing them from a distance, if at all. Naive human participants cannot reliably infer the emotion displayed by a chimpanzee, though they can perform at levels above chance^23^. Moreover, humans distinguish between happy (kept in social housing) macaque monkeys (genetically more closely related to humans than dogs, but with a less recent shared ancestor as panins) and unhappy macaques (kept in isolation), despite lacking the experience to support the inference from facial displays and having fewer facial and genetic similarities compared to panins^24^.

Core emotional circuits are common amongst mammals, including fear, rage, and play, and certain generalizable situations cause neurological patterns that are likely to correspond approximately to physiological patterns in all mammals, even humans^25^. Therefore, emotions are situationally determined where self-report is not available, such as in animals (see^26^, for a similar approach). For example, dogs display a fear response to veterinary treatment^27^, whilst panins often demonstrate fear of or submissive aversion to other, more dominant apes^28^. Both humans and panins find affiliative social contact positively reinforcing, eliciting joy or pleasure^29,30^, and play evokes pleasure in both panins and dogs^31,32^. Combat and conflict promote anger responses in the animal species^33,34^, and they have also been shown to dislike isolation^35,36^.

For this study, we chose emotions with the most robust scientific support for their analogous presence in dogs and panins; specifically, happiness or pleasure^33,37^, sadness or displeasure^36,38^, fear^28,39^, and anger^40,41^. The secondary rationale for choosing these facial displays is that they each have clearly defined emotional antecedents or contextual determinants. Finally, previous research has explored human inferences of emotion from facial displays in both species of animals^19,40,41^. Therefore, when considering the emotions of animals, it is reasonable to accept situationally defined approximations of well-established emotional responses. We do however acknowledge that animal facial displays may be indices of future behaviour rather than emotional experience^42,43^, and even human facial displays may not underlie true emotional experience^44^.

Lastly, inferring emotions from animals may engage anthropomorphism. Since humans perceive dogs as protective, high in warmth and competence, while chimpanzees as threatening, high in competence but low in warmth^45^, dogs and panins may be differently anthropomorphised.

Here, we directly test whether experience with or similarity to an animal matters for inferring emotion from their facial displays. Across two studies, participants perform an emotion categorisation task where they must select the correct emotion that corresponds to a facial display from humans, dogs, and panins. The presence of imitative brain systems: motor facilitation in the same muscles used by the person being observed^46^ and; similar activation patterns during observation and expression^47^ may allow for higher accuracy when inferring emotion from panin than dog facial displays. Conversely, recent exposure to members of a culture improved both the speed and accuracy of emotion inference of Chinese participants who lived in either China or the USA, regardless of participant’s own cultural background^48^. This suggests experience impacts inferring emotion from facial displays, which may allow for higher accuracy when inferring emotions from dog than panin facial displays. Further, we focus on animals’ differentiable facial displays in different contexts or when engaged in different behaviours. As such, we define accuracy as inference of the emotion of the animal consistent with the context or behaviour.

## Study 1: results

### Accuracy

We found a significant main effect of *species*, *Wald χ*^2^ (2) = 4319.42, *p* < .001, such that participants were most accurate for human faces (*M* = 0.95, *SE* = 0.01) compared to panin (*M* = 0.46, *SE* = 0.01; *LSD M*_*diff*_ = 0.49, *SE*_*diff*_ = 0.02, *p* < .001, *95% CI* [0.47, 0.50]) and dog faces (*M* = 0.60, *SE* = 0.01; *LSD M*_*diff*_ = 0.35, *SE*_*diff*_ = 0.02, *p* < .001, *95% CI* [0.33, 0.36]). However, participants were significantly more accurate for dog than panin faces; *LSD M*_*diff*_ = 0.14, *SE*_*diff*_ = 0.02, *p* < .001, *95% CI* [0.13, 0.16].

We also found a significant main effect of *emotion*, *Wald χ*^2^(3) = 271.39, *p* < .001, such that participants were most accurate for happy (*M* = 0.76, *SE* = 0.01) and neutral faces (*M* = 0.77, *SE* = 0.01) relative to sad (*M* = 0.56, *SE* = 0.01) and fearful inferences from faces (*M* = 0.60, *SE* = 0.01). Specifically, we failed to find a significant difference between accuracy for happy and neutral inference from faces; *LSD M*_*diff*_ = 0.01, *SE*_*diff*_ = 0.02, *p* = .431, *95% CI* [− 0.02, 0.04], and fear and sad inferences from faces; *LSD M*_*diff*_ = 0.05, *SE*_*diff*_ = 0.02, *p* = .002, *95% CI* [0.02, 0.08]. All other inferences from faces differed significantly on accuracy (all *p*’s < .001).

**Figure 1:**
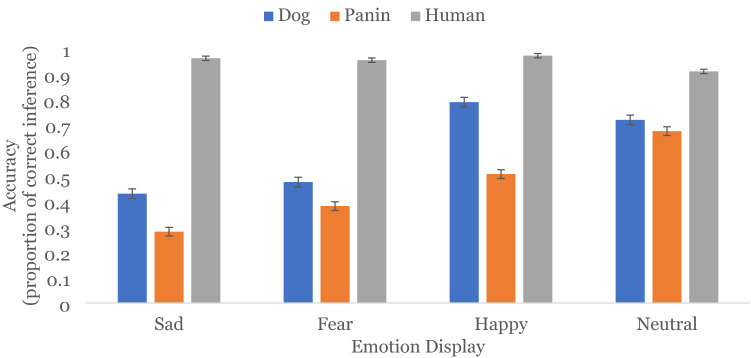
Proportion of accurate emotion inferences from facial displays in Study 1. Error bars represent standard error. All bars within emotion category are significantly different.

Both main effects were qualified by a significant *species X emotion* interaction, *Wald χ*^2^(6) = 471.63, *p* < .001 (see Fig. 1). To unpack this interaction, we consider comparisons that were not significant within species (see Table 1 for all simple effect results). Happy, sad, and fearful inferences from human faces (happy-sad *LSD M*_*diff*_ = 0.01 *SE* = 0.01, *p* = .203, *95% CI* [− 0.01, 0.03]; happy-fearful *LSD M*_*diff*_ = 0.02 *SE* = 0.01, *p* = .074, *95% CI* [− 0.00, 0.04]; sad-fearful *LSD, M*_*diff*_ = 0.01, *SE* = 0.01, *p* = .387, *95% CI* [− 0.01, 0.02]), did not differ in accuracy from each other, but all differed from neutral inferences from human faces, suggesting that participants performed equally well inferring emotion from human facial displays, but were less accurate inferring neutral from facial displays.

Sad and fearful inferences from dog faces (*LSD M*_*diff*_ = 0.05, *SE* = 0.03, *p* = .145, *95% CI* [− 0.22, 0.11]), and happy and neutral inferences from dog faces (*LSD M*_*diff*_ = 0.07, *SE* = 0.03, *p* = .005, *95% CI* [0.02, 0.12]) did not differ in accuracy, but participants were significantly better at inferring happy and neutral from dog faces from the two avoidant negative emotions. This suggests that participants struggled to infer negative avoidant emotions from facial displays in dog faces.

For panin faces, we found that participants were best at inferring neutral, then happy, then fearful, then sad from facial displays (all *p*’s < .001).

When further considering the *emotion* × *species* interaction, there is a consistent pattern of highest accuracy for inferences of emotion from human, then dog, then panin faces across all facial displays (all *p*’s < .001), except for neutral inferences from dog and panin faces which did not differ; *LSD M*_*diff*_ = 0.04, *SE*_*diff*_ = 0.02, *p* = .038, *95% CI* [0.002, 0.09].

### Reaction time

We found no significant main effects or interactions when exploring categorisation speed, suggesting participants may have found the task equally challenging across all conditions.

**Table 1:** Main effects and interaction on accuracy of emotion inferences across species and emotions in Study 1.

(I)	(J)	Mean diff. (I–J)	Std. error	Sig.	95% wald confidence interval for difference	
					Lower	Upper
**Emotion main effect**						
Happy	Sad	.199*	.013	> .001	.173	.225
Happy	Fear	.153*	.017	> .001	.119	.186
Fear	Sad	.046^	.015	.002	.017	.075
Neutral	Happy	.012	.016	.431	− .018	.043
Neutral	Sad	.211*	.018	> .001	.177	.246
Neutral	Fear	.165*	.015	> .001	.136	.194
**Species main effect**						
Dog	Bonobo/chimp	.143*	.009	> .001	.126	.160
Human	Dog	.346*	.008	> .001	.330	.362
Human	Bonobo/chimp	.489*	.008	> .001	.474	.504
**Emotion × Species interaction**						
*Dog comparisons across emotions*						
Happy	Sad	.360*	.024	> .001	.312	.407
Happy	Fear	.314*	.031	> .001	.255	.373
Happy	Neutral	.070^	.025	.005	.021	.119
Fear	Sad	.046	.031	.145	− .016	.107
Neutral	Sad	.290*	.030	> .001	.231	.348
Neutral	Fear	.244*	.027	> .001	.192	.296
*Bonobo/chimpanzee comparisons across emotions*						
Happy	Sad	.226*	.025	> .001	.177	.275
Happy	Fear	.018	.010	.074	− .002	.037
Fear	Sad	.101*	.021	> .001	.060	.141
Neutral	Happy	.169*	.029	> .001	.112	.225
Neutral	Sad	.395*	.027	> .001	.342	.448
Neutral	Fear	.295*	.021	> .001	.253	.336
*Human comparisons across emotions*						
Happy	Sad	.010	.008	.203	− .005	.026
Happy	Fear	.018	.010	.074	− .002	.037
Happy	Neutral	.062*	.011	> .001	.041	.083
Sad	Fear	.008	.009	.387	− .010	.025
Sad	Neutral	.052*	.012	> .001	.029	.074
Fear	Neutral	.044*	.012	> .001	.021	.067
*Happy comparisons across species*						
Human	Dog	.182*	.017	> .001	.149	.216
Human	Bonobo/chimp	.465*	.020	> .001	.427	.503
Dog	Bonobo/chimp	.283*	.022	> .001	.239	.327
*Sad comparisons across species*						
Human	Dog	.532*	.020	> .001	.493	.572
Human	Bonobo/chimp	.682*	.156	> .001	.651	.712
Dog	Bonobo/chimp	.149*	.020	> .001	.110	.189
*Fear comparisons across species*						
Human	Dog	.479*	.020	> .001	.440	.518
Human	Bonobo/chimp	.573*	.015	> .001	.544	.603
Dog	Bonobo/chimp	.095*	.019	> .001	.057	.132
*Neutral comparisons across species*						
Human	Dog	.191*	.019	> .001	.154	.227
Human	Bonobo/chimp	.235*	.019	> .001	.197	.273
Dog	Bonobo/chimp	.044^	.021	.038	.002	.085

*significant at Bonferroni corrected *p* < .0013; (^) marginally significant.

### Confidence

We found a significant *species* main effect, *F* (2, 292) = 229.62, *p* < .001, *η*_*p*_^2^ = 0.61, *Ω* = 1.00. Follow-up pairwise comparisons revealed a significant difference between humans and dogs (*M*_*diff*_ = 23.41, *SE* = 1.90, *p* < .001, *95% CI* [19.66, 27.16]), humans and panins (*M*_*diff*_ = 41.71, *SE* = 2.02, *p* < .001, *95% CI* [37.71, 45.72]), and dogs and panins (*M*_*diff*_ = 18.31, *SE* = 1.93, *p* < .001, *95% CI* [14.49, 22.12]). This suggests that participants were most confident inferring human facial displays, and least confident inferring panin facial displays, with dogs in the middle.

### Correlations

We found a significant correlation between experience and confidence for inferences of emotion from dog facial displays, *r* (143) = 0.29, *p* < .001, such that more experience was related to increased confidence. We did not observe a significant correlation for panins, *r* (143) = 0.11, *p* = .181.

We also found a significant correlation between dog experience and reaction time, *r* (143) = 0.38, *p* < .001 such that participants more experienced with dogs took longer to infer emotions from their facial displays. We did not observe a significant correlation for panins, *r* (143) = 0.01, *p* = .905. This perhaps is an artefact of the circumstances participants encounter the animals, with dog–human interactions likely to occur in domestic contexts, while panin-human interactions likely to occur in captive contexts.

We also found a significant correlation between dog, *r* (131) = 0.19, *p* = .032 and panin experience and accuracy, *r* (143) = 0.27, *p* = .001, such that participants more experienced with both species were more accurate at inferring emotion from their facial displays. This is consistent with both theorising and previous literature.

Finally, we also found a significant correlation between confidence in inferring human facial displays and reaction time, *r* (145) = − 0.22, *p* = .006, such that people who were more confident were faster to categorise human facial displays. We did not find significant correlations for confidence and reaction time for any other species (all *p*’s > .050). We also did not find significant correlations between confidence and accuracy for any species (all *p*’s > .050). This suggest that a meta-cognitive evaluation was only relevant for human faces, the species participants felt most confident categorising.

## Study 2

We followed Study 1 with a second study aimed at replicating the findings and broadening the emotion inferences. The most substantiative difference reduced the number of emotions to three, replacing fear and sadness with anger. Though anger is also a negatively valenced emotion, it is an approach emotion, and a conspecific with a facial display of anger and directed gaze is a threat, unlike one displaying sadness or fear. We kept most other aspects of the study the same (see the *Methods Section* for other minor differences).

## Results

### Accuracy scores

We found a significant main effect of *species*, *Wald χ*^2^ (2) = 529.85, *p* < .001, such that participants were most accurate for human faces (*M* = 0.79, *SE* = 0.02) compared to panin (*M* = 0.48, *SE* = 0.01; *LSD M*_*diff*_ = 0.30, *SE*_*diff*_ = 0.01, *p* < .001, *95% CI* [0.28, 0.33]) and dog faces (*M* = 0.61, *SE* = 0.02; *LSD M*_*diff*_ = 0.18, *SE*_*diff*_ = 0.01, *p* < .001, *95% CI* [0.15, 0.21]). However, participants were significantly more accurate for dog than panin faces, *LSD M*_*diff*_ = 0.12, *SE*_*diff*_ = 0.02, *p* < .001, *95% CI* [0.09, 0.15], replicating Study 1.

We also found a significant main effect of *emotion*, *Wald χ*^2^(2) = 29.59, *p* < .001, such that participants were more accurate inferring anger (*M* = 0.70, *SE* = 0.02) than happiness (*M* = 0.61, *SE* = 0.02) and neutral from facial displays (*M* = 0.57, *SE* = 0.02). We failed to find a significant difference between accuracy for happy and neutral inferences from faces; *LSD M*_*diff*_ = 0.03, *SE*_*diff*_ = 0.03, *p* = .197, *95% CI* [− 0.02, 0.09]. However, participants were more accurate inferring angry than happy (*LSD M*_*diff*_ = 0.09, *SE*_*diff*_ = 0.03, *p* = .001, *95% CI* [0.04, 0.14] and neutral from faces (*LSD M*_*diff*_ = 0.13, *SE*_*diff*_ = 0.02, *p* < .001, *95% CI* [0.08, 0.17]).

**Figure 2:**
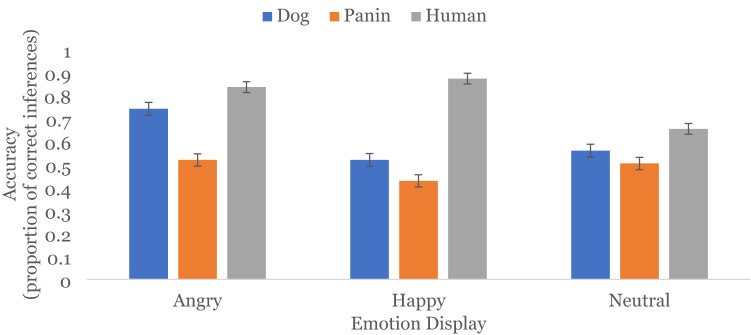
Proportion of accurate emotion inferences from facial displays in Study 2. Error bars represent standard error. All bars within emotion category are significantly different.

Both main effects were qualified by a significant *species* ×* emotion* interaction, *Wald χ*^2^(4) = 83.77, *p* < 0.001 (see Fig. 2). To unpack this interaction, we consider comparisons that were not significant within species (see Table 2 for all simple effect results). Accuracy rates for happy and angry inferences from human faces were not different (*LSD M*_*diff*_ = 0.04 *SE* = 0.02, *p* = .051, *95% CI* [0.00, 0.07]), but neutral inferences from human faces were significantly less accurate than both. This suggests that participants performed equally well when inferring emotions from human facial displays, but were less accurate when inferring neutral from facial displays, replicating the finding from Study 1.

**Table 2:** Main effects and interaction on accuracy of emotion inferences across species and emotions in Study 2.

(I)	(J)	Mean diff. (I–J)	Std. error	Sig.	95% wald confidence interval for difference	
					Lower	Upper
**Emotion main effect**						
Angry	Happy	.092*	.027	.001	.039	.145
Happy	Neutral	.034	.026	.197	− .018	.086
Angry	Neutral	.126*	.024	> .001	.080	.172
**Species main effect**						
Dog	Bonobo/chimp	.123*	.015	> .001	.093	.153
Human	Dog	.181*	.013	> .001	.155	.207
Human	Bonobo/chimp	.304*	.014	> .001	.277	.331
**Emotion × species interaction**						
*Dog comparisons across emotions*						
Angry	Happy	.222*	.045	> .001	.137	.308
Angry	Neutral	.182*	.032	> .001	.120	.244
Neutral	Happy	.040	.038	.285	− .034	.114
*Bonobo/chimpanzee comparisons across emotions*						
Angry	Happy	.091^	.042	.031	.008	.173
Neutral	Happy	.076	.041	.064	− .004	.156
Angry	Neutral	.015	.041	.666	− .054	.084
*Human comparisons across emotions*						
Happy	Angry	.037	.019	.051	− .0001	.073
Happy	Neutral	.218*	.027	> .001	.166	.271
Angry	Neutral	.182*	.028	> .001	.127	.237
*Happy comparisons across species*						
Human	Dog	.354*	.031	> .001	.294	.413
Human	Bonobo/chimp	.444*	.027	> .001	.391	.498
Dog	Bonobo/chimp	.091*	.028	.001	.037	.145
*Angry comparisons across species*						
Human	Dog	.095*	.028	.001	.041	.149
Human	Bonobo/chimp	.317*	.029	> .001	.260	.374
Dog	Bonobo/chimp	.222*	.027	> .001	.169	.276
*Neutral comparisons across species*						
Human	Dog	.095*	.023	> .001	.049	.141
Human	Bonobo/chimp	.150*	.025	> .001	.100	.200
Dog	Bonobo/chimp	.056^	.026	.036	.004	.107

*significant at Bonferroni corrected *p* < .0021; (^) marginally significant.

Inferences of happy and neutral from dog faces did not significantly differ in accuracy (*LSD M*_*diff*_ = 0.04, *SE* = 0.04, *p* = .285, *95% CI* [− 0.03, 0.11]), but participants were significantly more accurate inferring angry than happy or neutral from dog faces. This suggests that participants did not struggle to infer negative approach emotions from facial displays in dogs. Coupled with the results of Study 1, it suggests that positive approach emotions are better inferred from dog faces, though there seems to be a boost for anger—a negative approach emotion—potentially as a threat signal.

For panin faces, we found that accuracy did not differ when participants inferred angry and neutral, *LSD M*_*diff*_ = 0.02, *SE* = 0.04, *p* = 0.666, *95% CI* [− 0.05, 0.08], nor happy and neutral from faces, *LSD M*_*diff*_ = 0.08, *SE* = 0.04, *p* = 0.064, *95% CI* [0.00, 0.16]. nor anger and happy from faces, *LSD M*_*diff*_ = 0.09, *SE* = 0.04, *p* = .031, *95% CI* [0.01, 0.17].

When further considering the *species* × *emotion* interaction, there is a consistent pattern with participants showing the highest accuracy when inferring emotions from human, then dog, then panin faces across all facial displays (all *p*’s < .0021), except for neutral inferences from dog and panin faces, *LSD M*_*diff*_ = 0.06, *SE* = 0.03, *p* = .036, *95% CI* [0.004, 0.11], again replicating Study 1.

### Reaction times (RT)

There were no significant main effects or interactions for the reaction time measure, replicating the results of Study 1.

### Confidence

We found a significant *species* main effect, *F* (2, 262) = 13.59, *p* < .001, *η*_*p*_^2^ = 0.09, *Ω* = 1.00. Follow-up pairwise comparisons revealed a significant difference between confidence ratings when inferring emotions from humans and dogs (*M*_*diff*_ = 5.87, *SE* = 1.70, *p* = 0.001, *95% CI* [2.50, 9.24]), humans and panins (*M*_*diff*_ = 8.33, *SE* = 2.00, *p* < .001, *95% CI* [4.46, 12.20]), and dogs and panins (*M*_*diff*_ = 2.46, *SE* = 1.17, *p* = .037, *95% CI* [0.15, 4.76]). This suggests that participants were most confident inferring emotion from human facial displays followed by dog facial displays, and least confident inferring emotion from panin facial displays. These results replicate the findings in Study 1.

### Correlations

We found a significant correlation between dummy coded experience and confidence ratings for emotion inferences from panin faces, *r* (130) = 0.19, *p* = .027, as well as for dog faces, *r* (130) = 0.32, *p* < .001, such that more experience was related to increased confidence.

We found a significant correlation between dummy coded experience and reaction time when inferring emotion from dog facial displays, *r* (130) = 0.20, *p* = .020 such that participants who were more experienced with dogs took longer to infer emotion from their facial displays. The time experience variable did not significantly correlate with any other measures for dogs or panins, and we did not find a significant correlation between dummy coded experience and accuracy, reaction time, or confidence for emotion inferences from panin facial displays (all *p*’s > .050).

We also found a significant correlation between confidence in inferring dog facial displays and reaction time, *r* (130) = 0.32, *p* < .001, such that participants who were more confident of their inference also took longer. No other correlation between experience and accuracy reached significance when inferring emotion from dog facial displays, and there was no significant correlation between confidence and accuracy or reaction time (all *p*’s > .050) when inferring emotion from human facial displays.

## Discussion

The primary aim of this study was to investigate whether experience or similarity best explain inferring emotions from facial displays of non-human animals. To test this, we pitted a domesticated and experienced species with very different facial morphology to humans (dogs) against species with a more similar facial morphology but much less likely to be encountered by our participants (panins). We found evidence that participants were more accurate inferring dog than panin facial displays. Correlational evidence suggests that higher experience with either animal is associated with increased accuracy inferring emotion from that animals’ facial displays. This suggests that people are using their past experiences with the animal to infer emotions from facial displays.

We also explored emotion inference from human faces. In addition to better accuracy for human than animal faces, interestingly, we found that participants had more difficulty inferring neutral from human facial displays than the more emotional inferences; a difficulty they did not suffer with the animal inferences. This suggests a difference between how people infer emotions from human and animal faces, perhaps relying on more emotion-specific or mimicry mechanisms for human faces and learning mechanisms for animals. There may also be a predisposition towards inferring emotions from human facial displays that is not present for animals.

The pattern of results for the second study replicates the first as participants were more accurate inferring emotions from dog than panin facial displays. In addition, we found that there was no species advantage for inferring anger displays, consistent with an evolutionary preserved mechanism to detect threat. These results provide further support for the role of experience in inferring emotion from facial displays, though with the important caveat that when emotion inference is in service of threat perception, neither experience nor similarity modulate the effect.

Our findings replicate evidence in the literature demonstrating that humans can accurately categorise dog facial displays of emotion, perhaps because of experience with dogs, or changes in dogs’ facial morphology to facilitate communication with humans^16,18^. We find that participants inferred emotions from dogs faces with varying degrees of accuracy. Angry inferences were most accurate, and participants performed significantly better than for happy inferences from dogs. However, happy inferences were significantly more accurate than sad and fear inferences; both latter inferences were also significantly more accurate than neutral inferences. This suggests a bias towards inferring the face as a threat (angry) or safety (happy) stimulus, over emotional inferences related to the negative well-being of the animal (fear, sad).

We find that emotion inferences for panins were also comfortably above-chance levels, along with dogs and humans, with only sad inferences in Study 1 just above chance. Also in Study 1, we find evidence consistent with the literature showing that humans more accurately infer happy than sad from macaque faces^24^. This suggests a bias towards accurate inferences from the face as a safety signal. However, Study 2 showed no difference between any of the emotions, urging caution when interpreting the results for panins.

Caution interpreting panin results relative to dogs is also needed when examining the meta-cognitive confidence ratings from participants. They felt most confident in their human inferences, followed by dogs, then panins. Moreover, confidence was correlated with experience for dogs across both studies, and panins only in Study 2, suggesting that more familiarity with the species boosted meta-cognitive self-perceptions of performance. Confidence was also correlated negatively with human inference reaction time in Study 1, and positively correlated with dog inference reaction time in Study 2, suggesting different impacts of confidence on performance across the two species.

Across both studies we found support for an effect of experience on emotion inference; we found significant correlations between experience and accuracy for dog faces in both studies, and panins in Study 1. Experience also correlated with reaction time for dogs across both studies, but not panins, suggesting that experience impacts performance primarily for the domesticated animal.

We found that angry facial displays were recognized regardless of species, providing evidence for an evolutionarily preserved threat detection mechanisms regardless of similarity or experience. Interestingly we found that angry displays were marginally more accurate than neutral displays for panins, and all emotions were inferred less accurately relative to neutral displays for panins. This suggests that perhaps participants used their mirror system to simulate panin facial displays, reducing accuracy for the faces in emotional situations since these displays use different facial muscles than humans.

There are several limitations with our studies, beyond the debate regarding whether animals experience emotions. It is commonly known that dogs use other parts of the body, such as wagging their tail, to express their emotions^11^, and humans can even recognise the emotions of dogs by just listening to their barks^26^, suggesting that dog faces may not be the most prominent cue for emotion recognition. Indeed, though humans also rely on human body posture^49^ and prosody to convey and infer emotions^50^, dogs rely on body language to a greater extent than humans do^51^. Therefore, dog facial displays may not be the most prominent cue for emotion inference. We also did not design our study to test for participant gender differences, therefore we collected unequal samples of men and women in both studies.

In addition, facial displays in panins rely on different muscle configurations to communicate emotions than similar human emotional displays, despite the similar facial morphology^41^. Nonetheless, humans were better at recognising the emotions of dogs by merely looking at their faces. This clearly shows how experiences, like the amount of exposure to non-conspecific animals, override biological roots like homology of facial muscles when humans infer emotions in non-human animals. Additionally, we did not include all emotions in one study, so we cannot compare anger inferences with sad or fear. Finally, Study 1 is more complex than Study 2 because we include four emotion options rather than three.

We also conflated two types of experience in our study—experience with a domesticated versus captive animal. Participants in our sample were more likely to encounter domesticated dogs, and captive panins, adding a confound to our results. Finally, our participants read stories about dogs and chimpanzees before completing the emotion inference task, and rated them on several characteristics, which could have influenced their performance on the emotion inference task. However, this exploratory variable did not interact with any of our reported main effects or interactions, suggesting that this exploratory task did not differentially impact our participants.

Future research could investigate the role of species domesticity in our understanding of animal facial displays, as well as other factors which affect whether anthropomorphic thinking manifests as increased or decreased accuracy of judgements regarding animal behaviours. Future research could also explicitly manipulate the experience or similarity of an animal, and test whether these variables enhance emotion recognition from facial displays.

## Method

### Participants

#### Study 1

We recruited participants using an opportunity sample via social media advertisements and a psychology subject pool. Those who were recruited from the subject pool were awarded course credits for participation. There were no differences based on the source of participants. In total, 147 United Kingdom participants completed the study, comprised of 113 female and 31 male participants, as well as 3 participants who identified as other, between the ages of 18 and 71, *M*_*age*_ = 32.40 years, *SD*_*ag*e_ = 13.60. Post-hoc power analyses revealed that we had sufficient power to detect a medium effect size, *1−β* = 0.79. All participants were informed that they could leave the study at any time without consequence and gave their full informed consent before beginning the study. The study was approved by the University College London Ethics Committee; all experimental protocols were approved by this body, and all methods were carried out in accordance with relevant guidelines and regulations.

#### Study 2

We collected data from 132 United States participants via Amazon Mechanical Turk; 36 females and 96 males (*M*_*age*_ = 32.02 years, *SD*_*age*_ = 8.14, age range: 22–61 years); 51 White participants, 52 Asian participants, 16 Hispanic participants, and 13 Black other ethnicities participants. Post-hoc power analyses revealed that we had sufficient power to detect a medium effect size, *1−β* = 0.80. Participants were paid $1USD. We obtained ethical approval from the University College London Ethics Committee; all experimental protocols were approved by this body, and all methods were carried out in accordance with relevant guidelines and regulations.

### Materials and procedure

Across two studies, participants viewed facial displays associated with emotional situations or contexts and inferred the correct facial display to the associated emotion derived in that context. We used 8 images of each facial display for each species-emotion combination, for a total of 96 images in Study 1, and 6 images of each combination in Study 2, for a total of 54 images. We used equal numbers of males and females for human faces.

#### Study 1

We used Qualtrics to build the experiment. Participants viewed facial displays of three species (human, dog, panin), displaying emotions in four situations likely to evoke emotions (happy, sad, scared, neutral). There were 8 photographs for each category, so participants viewed 96 photos in total. The background of the photo was not visible; they were cropped so that only the face was visible to participants to avoid contextual information from the photo being used for emotion inference.

The photos of humans were used with permission from the Radboud database^52^. Only photos of people of phenotypically European descent were used (there was no race or ethnic diversity), and gender was balanced for each emotional category. The photographs of dogs were obtained with permission from a professional pet photographer (www.thedogphotographer.co.uk, 2018), and from Shutterstock Images (https://www.shutterstock.com, 2018), and showed a variety of breeds. Dog breeds in which head shape has been heavily selected, such as pugs, were avoided; instead, the photos were primarily of gundogs and mongrels. The number of dogs with erect and non-erect ears within each emotional category were balanced to prevent ear shape affecting the perception of the participants. Previous research depicted dogs in situations proven to evoke specific emotional responses, such as fear provoked by the presence of nail clippers^40^; therefore, the photos used in this study depicted a range of situations known to evoke specific emotions in most dogs to support the probable presence of the emotion (see Table 3).

This situation-emotion matching approach was also used when selecting photographs of panins (see Table 1). Expert evolutionary anthropologists have developed a facial coding system for chimpanzees (ChimpFACS^53^;) and they can discriminate between facial displays of chimpanzees^54^. One such experienced researcher provided photographs of bonobos and chimpanzees, and kindly categorized them into facial displays based on extensive experience of panin behaviour. These facial displays were then matched to likely emotions based on background information from the photo or photo source. No distinction was drawn between bonobos and chimpanzees (*Pan paniscus* and *Pan troglodytes*), and photos were not gender balanced for panins or dogs, as their facial sexual dimorphism, whilst present^55,56^, is not clearly visible to most humans.

**Table 3:** Contextual instantiation of emotion in non-human facial displays.

Species	Emotion	Situation	Relevant literature
Dogs	Happy	Playing with other dog/on a walk	Bekoff^31^
	Sad	Left tethered alone/stray in poor condition	Schwartz^36^
	Scared	Handled by a vet	Döring et al.^27^
Bonobos/Chimpanzees	Happy	Showing affiliative behaviour or play face	Steiner et al.^37^
	Sad	Isolated/in poor condition due to recent rescue	Alderton^38^
	Scared	Moving away from confrontation/scream face	Boesch^28^

Participants first read an anthropomorphic or mechanistic short story regarding a dog and a chimpanzee. Then, on seven-point Likert scale from ‘not at all’ to ‘very’, they rated chimpanzees on being *organised, neat, careful, in control of its actions*, and *sensitive* in the anthropomorphism condition*,* and *strong, easily hurt, large, dark coloured*, and *disciplined* in the mechanistic condition, while they rated dogs on *disciplined, lazy, thoughtful, easily hurt*, and *loyal* in the anthropomorphism condition, and *big, in control of its actions, furry, carnivorous*, and *sensitive* in the mechanistic condition. Those data were collected for exploratory reasons and will not be discussed further. Nonetheless, we tested whether this exploratory variable affected responses on the main dependent variable, and found no significant main effects of interactions (all *p*’s > .108 across both studies).

Participants clicked the link to the study to be directed to the website to begin. After reading an information sheet and filling in a consent form, they read the anthropomorphic or mechanistic short story followed by the rating questions.

They then saw the photos of the faces in a random order and indicated via mouse clicks which of the four emotions (happy, sad, fearful, neutral) best described the facial display in a forced choice format. There was no time limit. They then indicated their experience with dogs and chimpanzees by describing this experience (e.g. owned a dog for 4 years). We dummy coded these responses for analyses. Next, they rated their overall confidence in categorizing the faces from the three species on a Likert scale from 0 (no confidence) to 100 (very confident). They then completed an individual difference measure of flexible social cognition^57^ for exploratory purposes; we do not report the results of this measure. Finally, they provided demographic information, were debriefed, and thanked.

#### Study 2

We followed up the initial study with a second study that aimed to replicate these findings in a different sample. We used the same materials and followed the same procedure in Study 2 as Study 1 with a few exceptions. First, we reduced the number of emotions to three by eliminating sad and fearful and replacing them with anger: a negatively valenced approach emotion that serves as a threat signal when inferred in another. This allows us to determine whether all negatively valenced emotions are inferred the same way. Moreover, directed gazes displaying anger suggest that the entity with the face is a threat to the perceiver and therefore should trigger evolutionarily preserved threat-detection mechanisms.

Second, participants pressed a key to indicate their emotion choice rather than clicking the corresponding label on the screen. This change in response style facilitated more efficient and perhaps rapid responses. It also allows us to determine whether the null effects for reaction time are due to the response style or replicate across response style.

Third, we built the experiment with Gorilla experiment builder rather than Qualtrics, changing software platforms to rule out that the effect only occurred on a single online survey building platform.

Fourth, we asked about experience with the non-human animals by first having participants indicate the type of experience they had (dogs; dog owner, looked after dog, petted other people’s dog, other; chimpanzees; seen them in a zoo, watched them on TV, other) before indicating the amount of time they had this experience. We dummy coded types of experience as in Study 1 (dummy coded experience) and treated it as a separate variable from the amount of time (time experience). This change allowed us to get two experience dependent variables.

Fifth, we reduced the number of images per stimulus type from 8 to 6, resulting in a total of 54 images to shorten the length of the experiment. Finally, we asked participants to indicate which part of the face they paid most attention to when completing the task for each of the three species for exploratory reasons. All other procedures and materials remained the same. We kept all other elements of the design the same except where just noted.

### Data analysis strategy

The data analysis strategy remained the same across both studies. We used SPSS (V27) to analyse the data. The data were checked for normality using Box’s Test of Equality of Covariances of Means, and the reaction time data were transformed by Log10, therefore no outliers were removed from the reaction time or accuracy data. We converted accuracy to a proportion, such that 1.00 reflected perfect accuracy. We aggregated the data by averaging over the 8 trials in each of the 12 (Study 1) or 9 (Study 2) types of stimuli. To test the hypothesis that species affect categorisation accuracy, we completed Wald chi-square tests. This analysis deviated from our pre-registration of Study 2 where we stated we would perform parametric analyses. We computed a repeated measures ANOVA to investigate the effect of facial display of emotion and species on reaction time, and a second repeated measures ANOVA to determine whether participants expressed different levels of confidence in their ability to categorise the species. We corrected for multiple comparisons on the above inferential statistics with Bonferroni correction where appropriate. We used both Bonferroni corrected significance level and whether the confidence interval range included zero as criteria to determine statistical significance in our simple effect tests. Finally, we correlated experience, confidence, reaction time, and accuracy within species.

## Data Availability

The data is available at https://osf.io/n9z5q/. Study 2 is preregistered at https://aspredicted.org/MWX_SC1.

## References

[1] Kwan VS, Fiske ST (2008). Missing links in social cognition: The continuum from nonhuman agents to dehumanized humans. Soc. Cog..

[2] Gopnik A, Meltzoff AN (1997). Words, Thoughts, and Theories.

[3] Gopnik A, Wellman HM (1992). Why the child's theory of mind really is a theory. Mind Lang..

[4] Gordon RM (1986). Folk psychology as simulation. Mind Lang..

[5] Heal J, Carruthers P, Smith PK (1996). Simulation, Theory, and Content. Theories of Theories of Mind.

[6] De Vignemont F, Singer T (2006). The empathic brain: how, when and why?. Trends Cog. Sci..

[7] Gallese V (2001). The shared manifold hypothesis: From mirror neurons to empathy. J. Consci. Stud..

[8] Britten RJ (2002). Divergence between samples of chimpanzee and human DNA sequences is 5%, counting indels. Proc. Nat. Aca. Sci..

[9] Burrows AM, Waller BM, Parr LA, Bonar CJ (2006). Muscles of facial expression in the chimpanzee (*Pan*
*troglodytes*): Descriptive, comparative and phylogenetic contexts. J. Anat..

[10] Hare B, Tomasello M (2005). Human-like social skills in dogs?. Trends Cog. Sci..

[11] Bradshaw JW, Nott HM, Serpell J (1995). Social and Communication Behaviour of Companion Dogs. The Domestic Dog: Its Evolution, Behaviour and Interactions with People.

[12] Lycett SJ, Collard M, McGrew WC (2009). Cladistic analyses of behavioural variation in wild *Pan*
*troglodytes*: Exploring the chimpanzee culture hypothesis. J. Human Evo..

[13] Bekoff M (2007). The Emotional Lives of Animals: A Leading Scientist Explores Animal Joy, Sorrow, and Empathy—and why They Matter.

[14] Guo K, Tunnicliffe D, Roebuck H (2010). Human spontaneous gaze patterns in viewing of faces of different species. Perception.

[15] Kanwisher N, Stanley D, Harris A (1999). The fusiform face area is selective for faces not animals. NeuroReport.

[16] Wan M, Bolger N, Champagne FA (2012). Human perception of fear in dogs varies according to experience with dogs. PLoS ONE.

[17] Albuquerque N, Guo K, Wilkinson A, Savalli C, Otta E, Mills D (2016). Dogs recognize dog and human emotions. Bio. Lett..

[18] Kaminski J, Waller BM, Diogo R, Hartstone-Rose A, Burrows AM (2019). Evolution of facial muscle anatomy in dogs. Proc. Nat. Aca. Sci..

[19] Amici F, Waterman J, Kellermann CM, Karimullah K, Bräuer J (2019). The ability to recognize dog emotions depends on the cultural milieu in which we grow up. Sci. Rep..

[20] Van Hooff JARAM, Morris D (1967). The Facial Displays of the Catarrhine Monkeys and Apes. Primate Ethology.

[21] Hopkins WD, Taglialatela JP, Leavens DA, Vilain A, Schwartz J-L, Abry C, Vauclair J (2011). Do Chimpanzees have Voluntary Control of Their Facial Expressions and Vocalizations. Primate Communication and Human Language: Vocalisation, Gestures, Imitation and Deixis in Humans and non-Humans.

[22] Parr LA, Preuschoft S, de Waal FB, Ladygina-Kohts NN, de Waal FBM (2002). Afterword: Research on Facial Emotion in Chimpanzees, 75 years Since Kohts. Infant Chimpanzee and Human Child.

[23] Ekman P, Ekman P (1973). Cross-cultural Studies of Facial Expression. Darwin and Facial Expression: A Century of Research in Review.

[24] Gulledge JP, Fernández-Carriba S, Rumbaugh DM, Washburn DA (2015). Judgments of monkey’s (Macaca mulatta) facial expressions by humans: Does housing condition “affect” countenance?. Psych. Rec..

[25] Panksepp J (2005). Affective consciousness: Core emotional feelings in animals and humans. Consci. Cog..

[26] Pongrácz P, Molnár C, Miklósi A, Csányi V (2005). Human listeners are able to classify dog (Canis familiaris) barks recorded in different situations. J. Comp. Psych..

[27] Döring D, Roscher A, Scheipl F, Küchenhoff H, Erhard MH (2009). Fear-related behaviour of dogs in veterinary practice. Vet. J..

[28] Boesch C, Wrangham R, McGrew WC, de Waal FBM (1994). Hunting Strategies of Gombe and Tai chimpanzees. Chimpanzee Cultures.

[29] Mason WA, Saxon SV, Sharpe LG (1963). Preferential responses of young chimpanzees to food and social rewards. Psych. Rec..

[30] Trezza V, Baarendse PJ, Vanderschuren LJ (2010). The pleasures of play: Pharmacological insights into social reward mechanisms. Trends Pharm. Sci..

[31] Bekoff M (2004). Wild justice and fair play: Cooperation, forgiveness, and morality in animals. Bio. Phil..

[32] Leavens DA, Bard KA (2016). Tickling. Cur. Bio..

[33] Range F, Ritter C, Virányi Z (2015). Testing the myth: Tolerant dogs and aggressive wolves. Proc. Royal Soc. B: Bio. Sci..

[34] Wittig RM, Boesch C (2003). Decision-making in conflicts of wild chimpanzees: An extension of the relational model. Beh. Eco. and Sociobio..

[35] Reimers M, Schwarzenberger F, Preuschoft S (2007). Rehabilitation of research chimpanzees: Stress and coping after long-term isolation. Horm. Beh..

[36] Schwartz S (2003). Separation anxiety syndrome in dogs and cats. J. Am. Vet. Med. Assoc..

[37] Steiner JE, Glaser D, Hawilo ME, Berridge KC (2001). Comparative expression of hedonic impact: Affective reactions to taste by human infants and other primates. Neuro. Biobeh. Rev..

[38] Alderton D (2011). Animal Grief: How Animals Mourn.

[39] Hydbring-Sandberg E, von Walter LW, Hoglund K, Svartberg K, Swenson L, Forkman B (2004). Physiological reactions to fear provocation in dogs. J. Endocrin..

[40] Bloom T, Friedman H (2013). Classifying dogs’ (Canis familiaris) facial expressions from photographs. Behav. Process..

[41] Parr LA, Waller BM, Vick SJ, Bard KA (2007). Classifying chimpanzee facial expressions using muscle action. Emotion.

[42] Waller BM, Micheletta J (2013). Facial expression in nonhuman animals. Emot. Rev..

[43] Waller BM, Whitehouse J, Micheletta J (2017). Rethinking primate facial expression: A predictive framework. Neuro. Biobeh. Rev..

[44] Barrett LF, Adolphs R, Marsella S, Martinez AM, Pollak SD (2019). Emotional expressions reconsidered: Challenges to inferring emotion from human facial movements. Psych. Sci. Pub. Interest.

[45] Sevillano V, Fiske ST (2016). Animals as social objects. Euro. Psych..

[46] Gallese V, Goldman A (1998). Mirror neurons and the simulation theory of mind-reading. Trends Cog. Sci..

[47] Carr L, Iacoboni M, Dubeau MC, Mazziotta JC, Lenzi GL (2003). Neural mechanisms of empathy in humans: a relay from neural systems for imitation to limbic areas. Proc. Nat. Aca. Sci..

[48] Elfenbein HA, Ambady N (2003). When familiarity breeds accuracy: Cultural exposure and facial emotion recognition. J. Pers. Soc. Psych..

[49] Ambady N, Rosenthal R (1998). Nonverbal communication. Encyc. Mental Health.

[50] Scherer KR (2003). Vocal communication of emotion: A review of research paradigms. Speech Commun..

[51] Correia-Caeiro C, Guo K, Mills D (2021). Bodily emotional expressions are a primary source of information for dogs, but not for humans. Anim. Cog..

[52] Langner O, Dotsch R, Bijlstra G, Wigboldus DHJ, Hawk ST, van Knippenberg A (2010). Presentation and validation of the Radboud faces database. Cog. Emot..

[53] Vick SJ, Waller BM, Parr LA, Smith Pasqualini MC, Bard KA (2007). A cross-species comparison of facial morphology and movement in humans and chimpanzees using the facial action coding system (FACS). J. Nonverb. Beh..

[54] Parr LA (2003). The discrimination of faces and their emotional content by chimpanzees (Pan troglodytes). Ann. N.Y. Aca. Sci..

[55] Carrasco JJ, Georgevsky D, Valenzuela M, McGreevy PD (2014). A pilot study of sexual dimorphism in the head morphology of domestic dogs. J. Vet. Beh.: Clin. App. Res..

[56] Weston EM, Friday AE, Johnstone RA, Schrenk F (2004). Wide faces or large canines? The attractive versus the aggressive primate. Proc. R. Soc. London B Bio. Sci..

[57] Lantos D, Harris LT (2021). The humanity inventory: Developing and validating an individual difference measure of dehumanization propensity. J. Theor. Soc. Psych..

